# Cardiac baroreflex sensitivity during repeated handgrip exercise: Comparisons with sit‐stand maneuvers and spontaneous rest

**DOI:** 10.14814/phy2.70352

**Published:** 2025-05-05

**Authors:** Wenxing Qin, Marina Fukuie, Daisuke Hoshi, Shoya Mori, Tsubasa Tomoto, Shigehiko Ogoh, Jun Sugawara, Takashi Tarumi

**Affiliations:** ^1^ Institute of Health and Sport Sciences University of Tsukuba Tsukuba Ibaraki Japan; ^2^ Human Informatics and Interaction Research Institute National Institute of Advanced Industrial Science and Technology Tsukuba Ibaraki Japan; ^3^ Department of Biomedical Engineering Toyo University Saitama Japan; ^4^ Neurovascular Research Laboratory University of South Wales Pontypridd UK

**Keywords:** baroreflex, exercise, handgrip, sequence method, sit‐stand, transfer function

## Abstract

The cardiac baroreflex regulates arterial pressure via autonomic heart rate control. While sit‐stand maneuvers (SSM) have been used to assess baroreflex sensitivity (BRS), they may be impractical for physically immobile individuals. This study examined cardiac BRS during repeated handgrip exercise (RHE) compared to SSM and spontaneous rest. Twenty participants (10 females) performed 5‐min RHE and SSM at 0.10 and 0.05 Hz in random order after spontaneous rest. Cardiac BRS was calculated using transfer function analysis (BRS_TFA_) and the sequence method (BRS_SM_) in low (LF: 0.05–0.15 Hz) and very low (VLF: 0.02–0.07 Hz) frequencies. Power spectral density (PSD) quantified systolic blood pressure (SBP) and R‐R interval (RRI) oscillations. Compared to rest, 0.10 and 0.05 Hz RHE significantly increased SBP and RRI PSDs, with the highest values observed during SSM in both frequencies. RHE significantly increased LF and VLF BRS_TFA_ coherence by 132% and 142%, while SSM increased them by 144% and 209%. Regardless of analytical methods, BRS remained unchanged during RHE compared to rest, but it significantly decreased during 0.10 Hz SSM, which correlated with increased heart rate. These findings suggest that RHE improves BRS_TFA_ estimation via increased coherence, whereas reduced BRS during SSM suggests baroreflex resetting.

## 1 INTRODUCTION

Maintaining stable arterial blood pressure (ABP) is essential for ensuring continuous perfusion of vital organs in response to environmental stimuli encountered during daily activities (Ma et al., 2020). The baroreflex plays a crucial role in ABP regulation by detecting pressure‐induced changes in baroreceptors located in the aortic arch and carotid sinus (Fadel, 2008). The baroreflex triggers feedback control of heart rate (HR) and peripheral vascular resistance via the autonomic nervous system. Specifically, the cardiac baroreflex modulates HR in response to ABP fluctuations, with reduced baroreflex sensitivity (BRS) linked to advancing age and an increased risk of cardiovascular and neurological diseases (Dani et al., 2021; Ma et al., 2024).

Assessing cardiac BRS requires either ABP change or direct stimulation of arterial baroreceptors while simultaneously recording HR responses. At rest, spontaneous fluctuations in ABP and HR allow for a closed‐loop assessment of cardiac BRS. Alternatively, the BRS can be evaluated using pharmacological and non‐pharmacological interventions such as the modified Oxford method, neck chamber technique, and Valsalva maneuver (La Rovere et al., 2008). However, these approaches have limitations, including low clinical applicability, limited repeatability, reliance on specialized equipment, and/or the need for technical expertise (Raczak et al., 2001; Sleight et al., 1995). Recently, postural changes, such as repeated sit‐stand maneuvers (SSM) performed at low (LF: 0.10 Hz) and very low (VLF: 0.05 Hz) frequencies, are increasingly used as a non‐invasive and practical method for assessing cardiac BRS (Zhang et al., 2009). These maneuvers induce gravitational blood volume shifts and activate the exercise pressor reflex via lower‐body muscle contractions, leading to significant ABP fluctuations and baroreflex‐mediated HR responses. However, SSM may also elicit physiological responses similar to large‐muscle dynamic exercise, potentially resetting the baroreflex (Raven et al., 2006), and can be challenging for individuals with limited mobility or clinical conditions such as stroke.

Repeated handgrip exercise (RHE) has emerged as a novel alternative for assessing cardiac BRS. While static handgrip exercise increases mean ABP and HR, leading to baroreflex resetting (Raven et al., 2006), RHE performed at light intensity can generate oscillatory ABP changes at specific frequencies without significantly increasing mean HR (Qin et al., 2024). In previous research, the effects of static handgrip exercise on BRS have been studied, with limited exploration of “oscillatory” handgrip exercise for cardiac BRS assessment. Our recent study demonstrated that RHE performed at 30% of maximal voluntary contraction (MVC) can generate oscillatory ABP fluctuations in both LF and VLF ranges (Qin et al., 2024). These results suggest that RHE may be a viable method for assessing cardiac BRS, although this hypothesis remains to be tested.

Cardiac BRS can be quantified using the frequency‐domain or time‐domain methods. In the frequency domain, transfer function analysis (TFA) quantifies the magnitude (gain) and temporal (phase) relations between continuous ABP and HR oscillations, with the coherence function serving as a measure of linearity and signal‐to‐noise ratio for estimating cardiac BRS (Zhang et al., 2009). In the time domain, the sequence method can assess BRS by identifying spontaneous beat‐to‐beat changes in systolic blood pressure (SBP) and R‐R intervals (RRI). This approach enables the evaluation of cardiac BRS separately in upward and downward sequences, defined as three or more consecutive beats with parallel increases or decreases in SBP and RRI (Bagnall‐Hare et al., 2024).

Accumulating evidence suggests sex‐related differences in cardiac BRS. Studies indicate that females exhibit lower cardiovagal BRS than males, particularly under hypertensive stimuli, possibly due to sex‐specific variations in vagal activation patterns (Fu & Ogoh, 2019). Moreover, while males show an increase in cardiac BRS during isometric handgrip exercise, this response is absent in females (Samora et al., 2019). These findings suggest a potential interaction between sex and BRS, but the sex‐specific effects of RHE and SSM on cardiac BRS remain unexplored.

This study aimed to (Ma et al., 2020) determine whether RHE performed at light intensity can assess cardiac BRS and (Fadel, 2008) compare cardiac BRS among RHE, SSM, and spontaneous rest in young, healthy male and female participants. Using the TFA (BRS_TFA_) and sequence (BRS_SM_) methods, we hypothesized that RHE would enhance coherence for cardiac BRS_TFA_, accompanied by increased SBP and HR oscillations, while SSM would elicit the highest coherence and hemodynamic oscillations among all conditions. We further hypothesized that cardiac BRS_TFA_ and BRS_SM_ would be comparable across all conditions and that increased HR during SSM, but not during RHE, would be correlated with reduced cardiac BRS due to baroreflex resetting. Finally, based on prior research suggesting the sex‐specific effects on cardiac BRS (Fu & Ogoh, 2019; Samora et al., 2019), we hypothesized that BRS would be lower in females than in males at rest and during RHE and SSM.

## 2 METHODS

### 2.1 Participants

Participants of this study were recruited from our previous study that investigated the effect of RHE on dynamic cerebral autoregulation (Qin et al., 2024). Twenty participants (10 males and females) were included in this study. Individuals with a history of major medical conditions such as cardiovascular, cerebrovascular, kidney, and neurological diseases were excluded. Potential participants who smoked or had a history of smoking were excluded. No participants were taking cardiovascular‐acting medications (e.g., antihypertensive, antilipidemic, and antidiabetic agents). Based on self‐reported physical activity and medical history questionnaires (Herrmann et al., 2024), some participants were physically active but not training in the structured exercise program. All participants signed an informed consent after understanding the study protocol and procedures, which were explained in detail by investigators. This study complies with the standards set by the latest revision of the Declaration of Helsinki and was approved by the Institutional Review Boards of the National Institute of Advanced Industrial Science and Technology.

### 2.2 Study protocol and procedure

Before the laboratory visit, participants were instructed to fast for >3 h and abstain from alcohol, caffeinated beverages, and vigorous exercise for >24 h. Data collection was conducted in a quiet, environmentally controlled laboratory. Following the height and body mass measurements, baseline brachial ABP was recorded after >10 min of rest in a seated position. MVC was measured twice, with the higher value used to calculate %MVC for RHE trials. Participants were familiarized with RHE and SSM protocols while investigators provided detailed instructions and ensured the recording of clean physiological signals.

Following a 5‐min baseline data collection in the seated resting position, participants underwent four conditions in random order: RHE and SSM performed at 0.10 Hz (5‐s intervals) and 0.05 Hz (10‐s intervals) with each condition lasting for 5 min. RHE was performed at 30% MVC. Prerecorded audio files were used during RHE and SSM to coach participants on the timing of forearm contractions and postural changes. Real‐time handgrip force (%MVC) was displayed on a computer screen placed in front of participants during RHE. Between each condition, a recovery period of at least 5 min was given to ensure that all hemodynamic variables returned to the baseline levels. During rest and the four conditions, finger ABP, ECG, and respiratory rate were continuously recorded and used for data analysis. To facilitate uninterrupted data recording, the servo mechanism of the finger cuff was disabled during each condition. Participants breathed spontaneously and were instructed to avoid any Valsalva‐like maneuvers in each condition. After each condition, participants reported their rate of perceived exertion (RPE) using the Borg scale (Aengevaeren et al., 2013; Bagnall‐Hare et al., 2024; Fu & Ogoh, 2019; Herrmann et al., 2024; Parati et al., 1989; Qin et al., 2024; Raczak et al., 2001; Raven et al., 2006; Samora et al., 2019; Skow et al., 2022; Sleight et al., 1995; Sugawara et al., 2003; Tarumi & Zhang, 2023; Tomoto et al., 2021; Zhang et al., 2009).

### 2.3 Instrumentation

Participants performed RHE with the dominant arm using a grip force transducer (ADInstruments, Dunedin, New Zealand). HR was measured by a 3‐lead electrocardiogram (ECG). ABP was recorded from the non‐dominant arm, and a finger photoplethysmography cuff (Human NIBP Nano Interface, ADInstruments) was placed on the middle digit of the same hand. The non‐dominant forearm was stabilized at heart level by using an arm sling, ensuring a relaxed position throughout the protocol. Finger ABP recording collected using a finger photoplethysmography has been validated against the intra‐arterial recording at rest and during laboratory testing (Parati et al., 1989). The Modelflow method calculated beat‐to‐beat stroke volume (SV), cardiac output (CO), and total peripheral resistance (TPR) from finger ABP waveforms (Sugawara et al., 2003), using the non‐invasive cardiac output extension in LabChart (ADInstruments) to process and analyze the data. Brachial cuff ABP was measured between each condition, while finger ABP was continuously recorded throughout data collection. Respiratory rate and end‐tidal carbon dioxide (EtCO_2_) were continuously recorded by capnography using a nasal cannula (Nihon Kohden, Tokyo, Japan). All data were simultaneously recorded at a sampling frequency of 200 Hz using the LabChart v8 data acquisition and analysis software.

### 2.4 BRS_TFA_ and PSD

Spectral and transfer function analyses were conducted following the methods established in our previous studies (Tomoto et al., 2021). BRS_TFA_ was evaluated using three parameters: gain, phase, and coherence. Gain represents the magnitude relation between the input (SBP) and output (RRI) signals, while phase reflects their temporal difference. Coherence measures the strength of the linear relation between SBP and RRI, ranging from 0 (no correlation) to 1 (perfect linear correlation). The magnitude of SBP and RRI oscillations was quantified using power spectral density (PSD). TFA and PSD parameters were first plotted across the entire frequency range and subsequently extracted from low‐frequency (LF) and very low‐frequency (VLF) bands or point estimates. For RHE and SSM, point frequency estimates of 0.10 Hz and 0.05 Hz were used because hemodynamic oscillations were deliberately amplified at these specific frequencies. For spontaneous rest, LF (0.05–0.15 Hz) and VLF (0.02–0.07 Hz) bands were used, consistent with prior studies (Aengevaeren et al., 2013; Zhang et al., 2009). These bands were selected because frequency banding reduces random noise and enhances the reliability of spectral estimates (Tarumi & Zhang, 2023) and because the frequencies used for RHE and SSM align with the centers of these LF and VLF bands.

### 2.5 BRS_SM_ and baroreflex effectiveness index (BEI)

BRS_SM_ was calculated using Nevrokard BRS analysis software (Nevrokard, Izola, Slovenia), as previously described (Skow et al., 2022). Sequences of three or more consecutive beats with SBP and RRI changes in the same direction were identified using the default software settings: a one‐beat delay, an *R* ^2^ inclusion criterion of 0.85, and thresholds of 1.0 mmHg for SBP and 5 ms for RRI. BRS_SM_ was categorized into overall, up‐, and down‐BRS_SM_. The overall BRS_SM_ represents the average value, including both up‐ and down‐sequences, while up‐ and down‐BRS_SM_ specifically reflect BRS during periods of increasing and decreasing SBP and RRI, respectively (Steptoe & Vogele, 1990). To assess how effectively the baroreflex translates spontaneous ABP fluctuations into HR adjustments, the baroreflex effectiveness index (BEI) was calculated using the following formula (Di Rienzo et al., 2001):

BEI=Number ofSBPsequences/Number ofSBPramps

where SBP ramps are defined as three or more consecutive beats in which SBP increases or decreases consistently.

### 2.6 Sample size estimate

The sample size estimate for this study is based on a previous study that measured cardiac BRS_TFA_ during spontaneous rest and SSM in 10 healthy young participants (Zhang et al., 2009). This study reported increases in coherence function from spontaneous rest to SSM in LF (rest vs. SSM: 0.65 ± 0.09 vs. 0.97 ± 0.03, effect size = 4.03) and VLF (rest vs. SSM: 0.38 ± 0.11 vs. 0.98 ± 0.01, effect size = 5.69). Based on these means, standard deviations (SD), and estimated effect sizes, at least four participants were expected to provide 95% power in detecting a significant change in coherence between the resting and SSM conditions with an *α* level of <0.05 (G*Power, Heinrich Heine University Düsseldorf, Northrhine‐Westphalia, Germany). Because ABP and HR oscillations during RHE may be smaller than during SSM, we enrolled 20 participants in the current study and explored sex‐related differences in cardiac BRS.

### 2.7 Statistical analysis

The normality of continuous variables was examined by the Shapiro–Wilk test and visual inspections of the histogram and Q‐Q plot. Mixed analysis of variance (ANOVA) analyzed the main and interaction effects of condition (within‐subject factors: RHE, SSM, rest) and sex (between‐subject factors: male and female). Additionally, one‐way repeated‐measures ANOVA compared the number of sequences between each condition. RPE was compared via the non‐parametric Friedman test. Mauchly's test of sphericity was performed for mixed ANOVA, and if the assumption of sphericity was violated, the Huynh‐Feldt correction was applied to adjust the degrees of freedom. Post‐hoc pairwise comparisons were performed with the Bonferroni correction for multiple comparisons if significant effects of condition (rest vs. RHE vs. SSM) or condition*sex interaction were found from mixed ANOVA. Simple correlations between continuous variables were examined by the Pearson product–moment correlation. Statistical significance was set a priori at *p* <0.05. All results are expressed as means ± standard deviations. All statistical analyses were performed by using the SPSS software (version 28.0, IBM Corporation, Armonk, NY).

## 3 RESULTS

### 3.1 Participant characteristics

Participants had a mean age of 31 ± 7 years, with no significant age difference between males (31 ± 5 years) and females (31 ± 9 years). All participants were normotensive, with similar SBP (130 ± 23 mmHg vs. 113 ± 11 mmHg, respectively) and BMI (22.4 ± 1.9 kg/m^2^ vs. 22.2 ± 3.2 kg/m^2^; *p* > 0.05) between males and females. However, diastolic blood pressure (DBP) was higher in males than in females (76 ± 17 mmHg vs. 61 ± 7 mmHg, *p* = 0.017). Maximal handgrip force was greater in males (43 ± 9 kg) than in females (20 ± 5 kg, *p* = 0.046). Males were taller (175 ± 6 cm) and weighed more (68 ± 8 kg) than females (161 ± 6 cm, 58 ± 9 kg).

### 3.2 Hemodynamic and respiratory measures

Table 1 summarizes the hemodynamic and respiratory data averaged over 5 minutes of spontaneous rest, RHE, and SSM in males and females. Significant condition effects were observed across all hemodynamic and respiratory measures. HR remained stable between rest and RHE but increased significantly during SSM, particularly at 0.10 Hz. A similar trend was observed for RRI. SBP increased during both RHE and SSM compared to rest, with the highest values recorded during SSM at 0.10 Hz. Males had higher SBP during RHE than females. Mean arterial pressure (MAP) increased during both RHE and SSM relative to rest, with males exhibiting higher MAP than females at rest and during RHE. DBP also increased during RHE, with a significant sex effect (higher in males). During SSM, SV and CO were higher while TPR was lower compared to rest and RHE. Respiratory frequency increased during RHE and SSM but remained outside the range for BRS_TFA_ estimation. EtCO_2_ was highest during SSM at 0.10 Hz. RPE was similar across all conditions.

**TABLE 1:** Hemodynamic and respiratory measurements during spontaneous rest, repeated handgrip exercise (RHE), and sit‐stand maneuvers (SSM) in young healthy male and female participants.

	Rest	RHE		SSM		*p‐*Value		
		0.10 Hz	0.05 Hz	0.10 Hz	0.05 Hz	Condition	Sex	Condition*sex
HR (bpm)								
All	70 ± 13	73 ± 12	73 ± 13	88 ± 14*, **, ***	81 ± 14*, **	<0.001	0.987	0.479
Male	69 ± 10	74 ± 10	73 ± 11	87 ± 14	81 ± 14			
Female	70 ± 16	72 ± 13	72 ± 15	89 ± 15	81 ± 14			
RRI (s)								
All	0.88 ± 0.16	0.84 ± 0.13	0.84 ± 0.14	0.70 ± 0.11*, **, ***	0.76 ± 0.12*, **	<0.001	0.899	0.354
Male	0.88 ± 0.13	0.82 ± 0.12	0.83 ± 0.13	0.70 ± 0.11	0.76 ± 0.12			
Female	0.89 ± 0.19	0.85 ± 0.15	0.85 ± 0.15	0.69 ± 0.11	0.76 ± 0.12			
SBP (mmHg)								
All	122 ± 19	134 ± 19*	133 ± 20*	144 ± 26*, **	137 ± 22*	<0.001	0.063	0.010
Male	130 ± 23	144 ± 22****	143 ± 21****	154 ± 33	141 ± 28			
Female	113 ± 11	125 ± 10	122 ± 12	135 ± 13	133 ± 13			
DBP (mmHg)								
All	68 ± 15	76 ± 16*	76 ± 14*	72 ± 17	72 ± 15	<0.001	0.034	<0.001
Male	76 ± 17****	83 ± 19****	85 ± 15****	79 ± 20	75 ± 20			
Female	61 ± 7	68 ± 7	67 ± 6	65 ± 10	68 ± 7			
MAP (mmHg)								
All	86 ± 15	94 ± 16*	94 ± 15*	94 ± 19*	91 ± 16*	<0.001	0.055	<0.001
Male	93 ± 18****	101 ± 19****	102 ± 16****	101 ± 23	94 ± 21			
Female	79 ± 7	87 ± 8	86 ± 7	86 ± 11	88 ± 8			
SV (mL)								
All	70.8 ± 20.1	70.7 ± 22.0	67.8 ± 17.1	83.5 ± 18.2*, **	77.6 ± 22.1**	<0.001	0.885	0.094
Male	69.0 ± 23.0	71.6 ± 27.4	66.1 ± 19.4	85.6 ± 20.1	81.2 ± 27.9			
Female	72.6 ± 17.6	69.7 ± 16.5	69.4 ± 15.4	81.4 ± 16.9	74.1 ± 15.1			
CO (L/min)								
All	4.9 ± 1.5	5.1 ± 1.6	4.9 ± 1.4	7.3 ± 1.9*, **, ***	6.2 ± 1.7*, **	< 0.001	0.934	0.249
Male	4.7 ± 1.2	5.2 ± 1.6	4.8 ± 1.1	7.3 ± 1.6	6.4 ± 1.8			
Female	5.1 ± 1.8	5.1 ± 1.8	5.1 ± 1.7	7.3 ± 2.2	6.0 ± 1.6			
TPR (mmHg·min/L)								
All	19.6 ± 9.0	20.6 ± 9.2	21.1 ± 8.6	14.0 ± 6.1*, **, ***	16.3 ± 7.3*, **	<0.001	0.364	0.014
Male	22.2 ± 10.7	22.3 ± 10.9	23.5 ± 10.0	15.1 ± 6.9	16.8 ± 8.8			
Female	17.1 ± 6.5	18.9 ± 7.3	18.7 ± 6.5	13.0 ± 5.3	15.9 ± 5.9			
RF (Hz)								
All	0.27 ± 0.03	0.30 ± 0.04*	0.29 ± 0.04*	0.30 ± 0.06	0.30 ± 0.03*	<0.001	0.620	0.395
Male	0.27 ± 0.03	0.30 ± 0.04	0.30 ± 0.04	0.28 ± 0.05	0.30 ± 0.02			
Female	0.27 ± 0.03	0.30 ± 0.03	0.29 ± 0.03	0.31 ± 0.06	0.31 ± 0.04			
EtCO_2_ (mmHg)								
All	35 ± 3	35 ± 2	35 ± 3	38 ± 3*, **, ***	36 ± 3**	<0.001	0.021	0.835
Male	37 ± 3	36 ± 3	36 ± 2	39 ± 3	37 ± 2			
Female	34 ± 2	34 ± 2	34 ± 2	36 ± 3	35 ± 2			
RPE								
All	–	11 ± 3	10 ± 3	10 ± 2	9 ± 2	0.072	0.521	0.962
Male	–	11 ± 3	11 ± 3	10 ± 2	9 ± 2			
Female	–	10 ± 2	10 ± 3	10 ± 2	9 ± 2			

*Note*: Values are means ± standard deviations in 10 males and 10 females. *p*‐values for continuous variables were calculated by mixed analysis of variance (ANOVA).

Abbreviations: CO, cardiac output; DBP, diastolic blood pressure; EtCO_2_, end tidal CO_2_; HR, heart rate; RF, respiratory frequency; RPE, rate of perceived exertion; RRI, R‐R interval; SBP, systolic blood pressure; SV, stroke volume; TPR, total peripheral resistance.

*p < 0.05 vs. rest, **p < 0.05: RHE vs. SSM in the same frequency, ***p < 0.05: 0.10 Hz vs. 0.05 Hz within the RHE or SSM condition, ****p < 0.05: male vs. female.

### 3.3 PSD analysis

**FIGURE 1:**
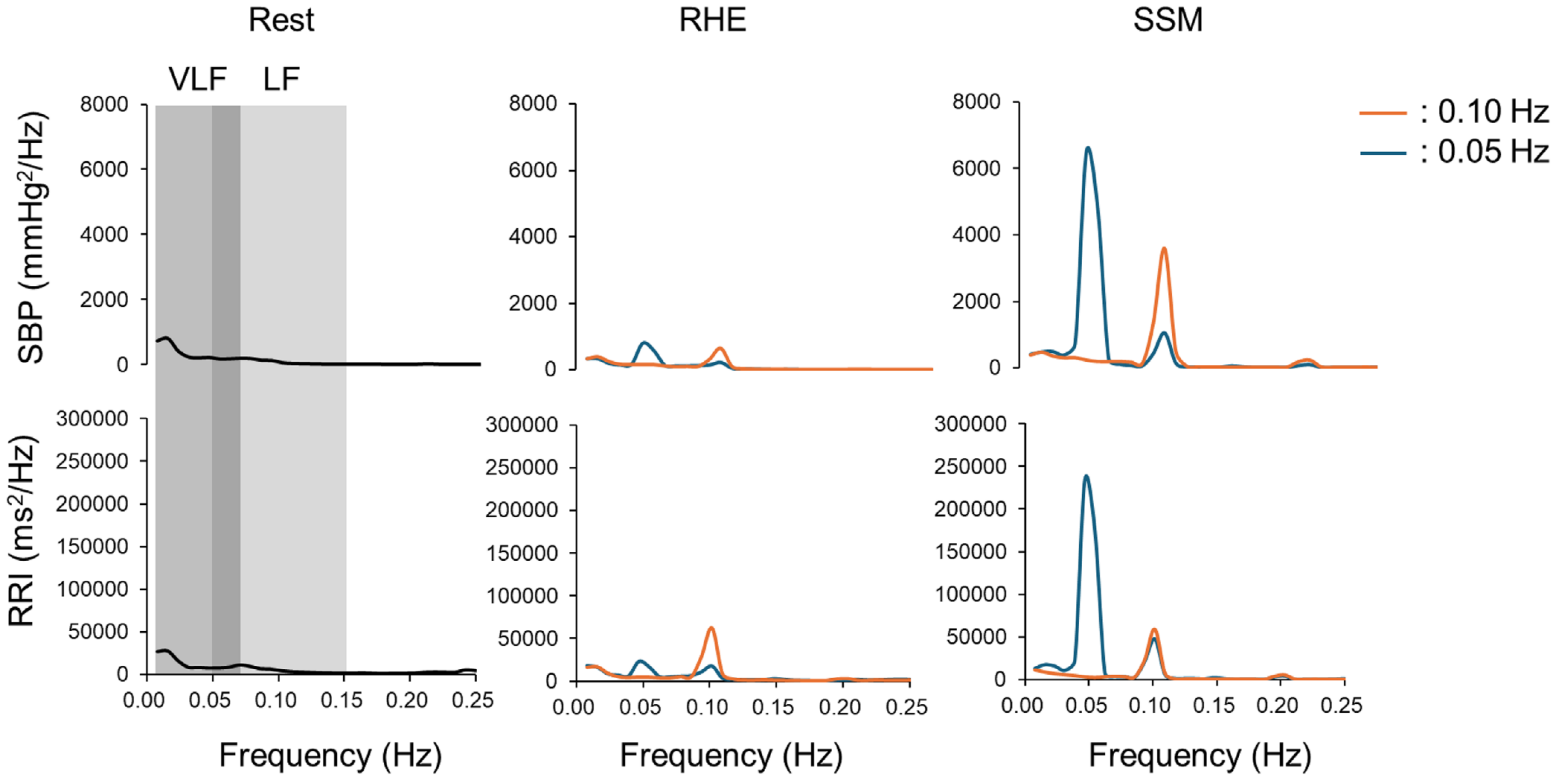
Group‐averaged power spectral density (PSD) plots of systolic blood pressure (SBP) and R‐R interval (RRI) during spontaneous rest, repeated handgrip exercise (RHE), and sit‐stand maneuvers (SSM) at 0.10 and 0.05 Hz. Data from 20 participants (10 males and 10 females) are included in theanalysis.

**FIGURE 2:**
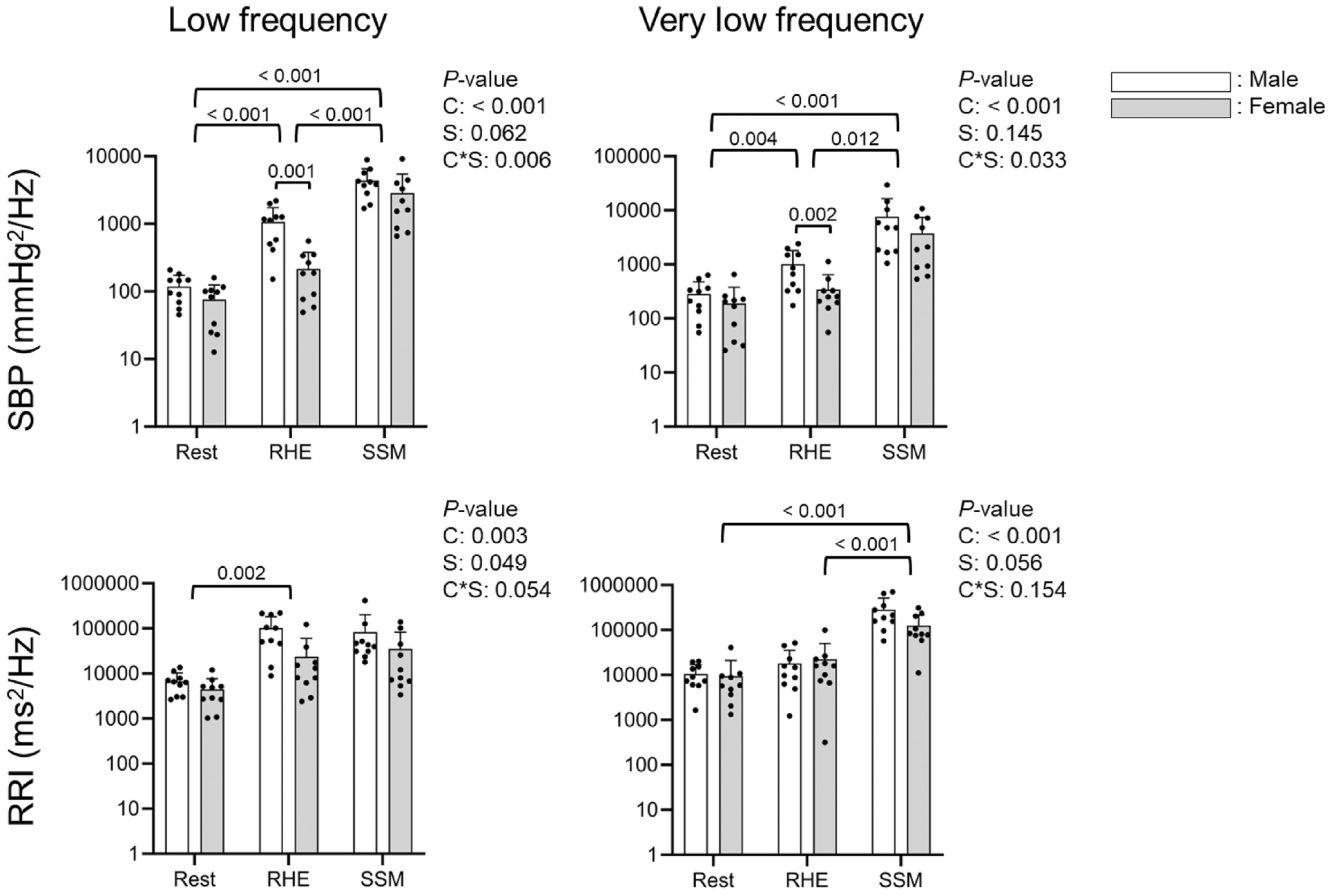
Power spectral densities of systolic blood pressure (SBP) and R‐R interval (RRI) compared among spontaneous rest, repeated handgrip exercise (RHE), and sit‐stand maneuvers (SSM) by sex. Y‐axes are log‐scaled. Point estimates are used for RHE and SSM (0.10 and 0.05 Hz for low and very low frequencies), while frequency bands are used for spontaneous rest (0.05–0.15 Hz and 0.02–0.07 Hz for low and very low frequencies). Two‐way mixed analysis of variance (ANOVA) compared the three conditions in 10 males and 10 females, with the Bonferroni correction for post‐hoc pairwise comparisons. Reported *p*‐values include main effects of condition (C), sex (S), and their interaction (C*S). Significant *p*‐values from post‐hoc tests are shown in the figure.

Figure 1 shows the PSD waveforms of SBP and RRI across the full frequency range during rest, RHE, and SSM in all participants. PSD peaks were visually identified at 0.05 and 0.10 Hz during RHE and SSM conditions, with greater peaks during SSM than during rest or RHE. Statistical analysis confirmed significant condition effects across all PSD measures (Figure 2). In LF, SBP PSD was significantly higher during RHE compared to rest (641 ± 644 vs. 97 ± 55 mmHg^2^/Hz, *p* <0.001) but lower than during SSM (3598 ± 2455 mmHg^2^/Hz, *p* < 0.001). In VLF, SBP PSD increased from rest to RHE (679 ± 670 vs. 237 ± 191 mmHg^2^/Hz, *p* = 0.004) but remained lower than during SSM (5666 ± 6842 mmHg^2^/Hz, *p* = 0.012). For RRI, PSD in LF was significantly greater during RHE than at rest (62,559 ± 73,836 vs. 5571 ± 3524 ms^2^/Hz, *p* = 0.002) and showed a trend toward higher values during SSM (58,941 ± 91,929 ms^2^/Hz, *p* = 0.051). In VLF, RRI PSD was highest during SSM (204,666 ± 185,316 ms^2^/Hz, *p* < 0.001 vs. rest and *p* < 0.001 vs. RHE). Significant interactions between condition and sex were observed for SBP PSD in both LF and VLF, with males exhibiting higher values than females during RHE (*p* = 0.001 and *p* = 0.020, respectively).

### 3.4 BRS_TFA_ analysis

**FIGURE 3:**
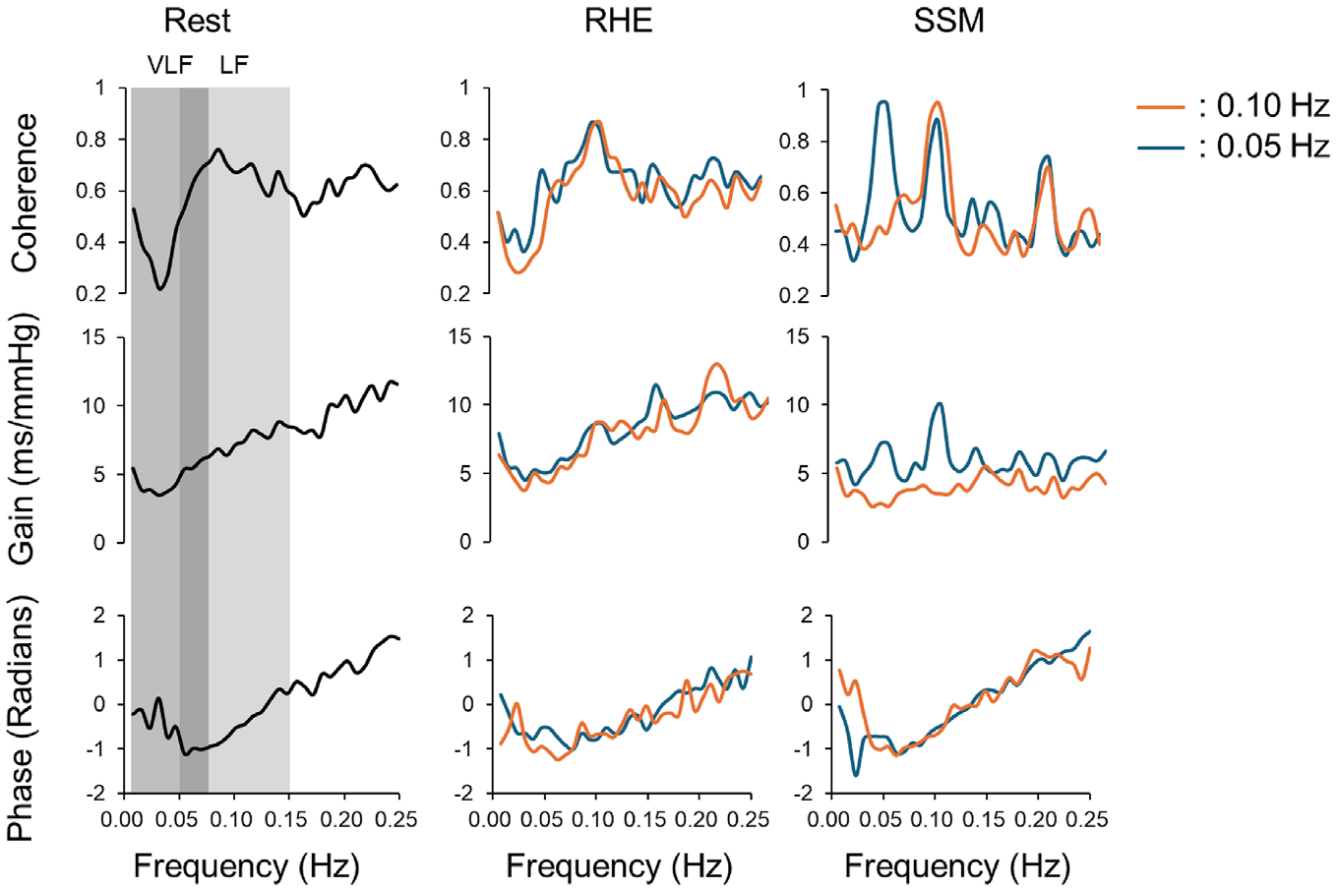
Group‐averaged waveforms of transfer function coherence, gain, and phase of the cardiac baroreflex sensitivity (BRS_TFA_) during spontaneous rest, repeated handgrip exercise (RHE), and sit‐stand maneuvers (SSM) performed at 0.10 and 0.05 Hz. Data from 20 participants (10 males and 10 females) are included.

**FIGURE 4:**
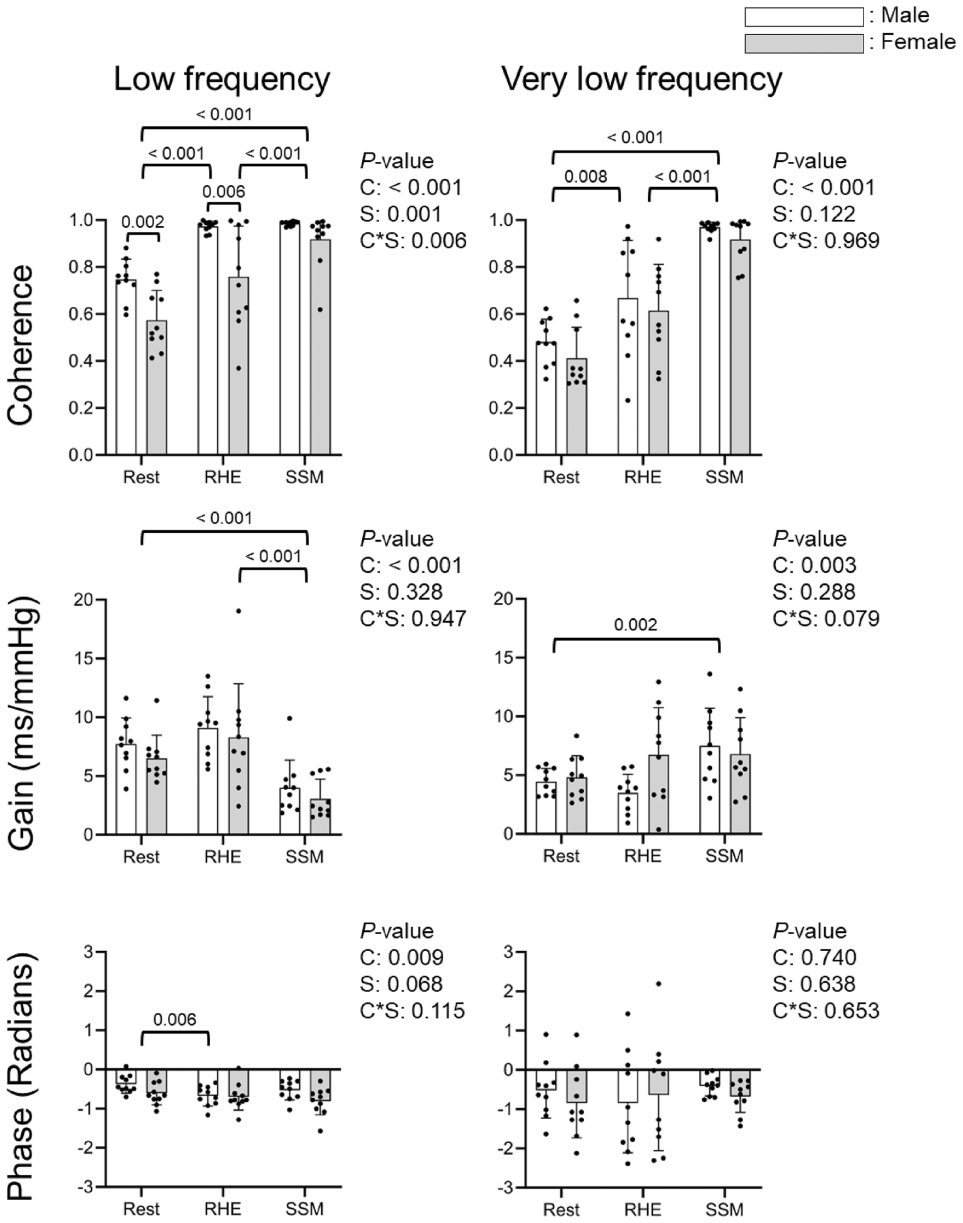
Transfer function coherence, gain, and phase of the cardiac baroreflex sensitivity (BRS_TFA_) compared among spontaneous rest, repeated handgrip exercise (RHE), and sit‐stand maneuvers (SSM) conditions by sex. Point estimates are used for RHE and SSM (0.10 Hz and 0.05 Hz for low and very low frequencies), while frequency bands are used for spontaneous rest (0.05–0.15 Hz and 0.02–0.07 Hz for low and very low frequencies). Two‐way mixed analysis of variance (ANOVA) compared the three conditions in 10 males and 10 females, with the Bonferroni correction for post‐hoc pairwise comparisons. Reported *p*‐values include main effects of condition (C), sex (S), and their interaction (C*S). Significant *p*‐values from post‐hoc tests are shown in the figure.

Figure 3 presents the group‐averaged BRS_TFA_ coherence, gain, and phase waveforms during rest, RHE, and SSM in all participants. Visually, RHE and rest produced similar coherence patterns, whereas SSM exhibited distinct coherence peaks at 0.05 and 0.10 Hz, accompanied by reduced gain at 0.10 Hz. Statistical analysis confirmed significant condition effects on all BRS_TFA_ measures, except for VLF phase (Figure 4). Coherence was significantly higher during RHE than rest in LF (0.87 ± 0.19 vs. 0.66 ± 0.14, *p* < 0.001) and VLF (0.64 ± 0.22 vs. 0.45 ± 0.12, *p* = 0.008). The highest coherence values were observed during SSM in both LF (0.95 ± 0.09) and VLF (0.94 ± 0.07, *p* < 0.001 vs. all conditions).

BRS_TFA_ gain in LF was similar between RHE (8.7 ± 3.7 ms/mmHg) and rest (7.1 ± 2.1 ms/mmHg) but was significantly reduced during SSM (3.5 ± 2.0 ms/mmHg, both *p* < 0.001 vs. RHE and rest). In VLF, gain was comparable between RHE and rest but increased during SSM (7.1 ± 3.1 ms/mmHg, *p* = 0.002 vs. rest). BRS_TFA_ phase in LF was significantly lower during RHE (−0.68 ± 0.29 radians) than during rest (−0.49 ± 0.28 radians, *p* = 0.006), whereas no significant differences were observed in VLF. A significant interaction between condition and sex was observed for LF coherence, with males exhibiting higher coherence than females at rest (*p* = 0.002) and during RHE (*p* = 0.006).

### 3.5 BRS_SM_ and BEI

SSM at 0.10 Hz elicited a greater number of baroreflex sequences (26 ± 12) compared to RHE (15 ± 13) and rest (9 ± 8), with similar trends observed at 0.05 Hz (*p* < 0.05 for SSM vs. RHE and rest). Significant condition effects were observed for overall and down‐BRS_SM_ in LF and VLF (Table 2). In LF, overall BRS_SM_ was lower during SSM compared to rest (*p* = 0.008) and RHE (*p* < 0.001), with lower down‐BRS_SM_ during SSM compared to rest (*p* = 0.046). In VLF, overall BRS_SM_ was also lower during SSM compared to rest (*p* = 0.025). A significant interaction between condition and sex was observed for overall BRS_SM_ during RHE in LF, with males showing lower values than females (*p* = 0.045).

**TABLE 2:** Baroreflex sensitivity (BRS) and baroreflex effectiveness index (BEI) analyzed by the sequence method during rest, repeated handgrip exercise (RHE), and sit‐stand maneuvers (SSM) in young healthy male and female participants.

	Rest	RHE	SSM	*p‐*Value		
				Condition	Sex	Condition*sex
*Low frequency*						
BRS (ms/mmHg)						
Overall						
All	11.9 ± 7.9	10.3 ± 4.3	6.2 ± 2.9*, **	<0.001	0.312	0.033
Male	10.8 ± 7.1	8.4 ± 1.7***	6.3 ± 3.2			
Female	13.0 ± 8.9	12.2 ± 5.3	6.2 ± 2.7			
Up						
All	10.5 ± 6.9	9.7 ± 6.0	6.4 ± 3.2	0.067	0.544	0.734
Male	10.0 ± 6.3	8.6 ± 1.2	6.3 ± 3.2			
Female	11.2 ± 8.0	11.1 ± 9.1	6.5 ± 3.3			
Down						
All	13.8 ± 10.3	9.4 ± 4.0	6.7 ± 5.5*	0.027	0.295	0.289
Male	10.8 ± 8.0	8.2 ± 2.9	7.2 ± 7.0			
Female	16.5 ± 11.8	10.5 ± 4.8	6.1 ± 4.2			
BEI						
Overall						
All	0.17 ± 0.10	0.29 ± 0.23*	0.44 ± 0.18*^+^	<0.001	< 0.001	0.058
Male	0.21 ± 0.12	0.44 ± 0.25	0.51 ± 0.14			
Female	0.13 ± 0.07	0.14 ± 0.08	0.37 ± 0.18			
Up						
All	0.22 ± 0.15	0.31 ± 0.24	0.48 ± 0.22*	0.003	0.008	0.123
Male	0.27 ± 0.17	0.44 ± 0.25	0.53 ± 0.20			
Female	0.16 ± 0.09	0.14 ± 0.10	0.42 ± 0.24			
Down						
All	0.16 ± 0.10	0.33 ± 0.23*	0.39 ± 0.21*	< 0.001	0.002	0.047
Male	0.18 ± 0.11	0.51 ± 0.20***	0.50 ± 0.19***			
Female	0.13 ± 0.08	0.18 ± 0.11	0.30 ± 0.18			
*Very low frequency*						
BRS (ms/mmHg)						
Overall						
All	11.8 ± 8.1	10.4 ± 4.8	8.5 ± 3.3**	0.031	0.251	0.182
Male	10.8 ± 7.1	8.6 ± 2.6	7.9 ± 2.9			
Female	12.9 ± 9.5	12.4 ± 6.0	9.1 ± 3.8			
Up						
All	10.2 ± 6.9	9.5 ± 5.4	8.4 ± 3.1	0.444	0.323	0.290
Male	10.0 ± 6.3	7.6 ± 2.3	8.0 ± 2.9			
Female	10.5 ± 8.0	11.9 ± 7.2	8.9 ± 3.5			
Down						
All	12.4 ± 9.0	10.0 ± 5.6	8.0 ± 4.0	0.020	0.218	0.373
Male	10.7 ± 8.0	8.2 ± 3.3	7.4 ± 2.7			
Female	14.1 ± 10.0	11.9 ± 6.9	8.7 ± 5.0			
BEI						
Overall						
All	0.18 ± 0.10	0.21 ± 0.15	0.42 ± 0.14*, **	<0.001	0.015	0.573
Male	0.21 ± 0.12	0.27 ± 0.18	0.48 ± 0.10			
Female	0.14 ± 0.06	0.15 ± 0.06	0.34 ± 0.13			
Up						
All	0.23 ± 0.14	0.28 ± 0.21	0.45 ± 0.15*, **	0.005	0.051	0.534
Male	0.27 ± 0.17	0.35 ± 0.24	0.47 ± 0.13			
Female	0.17 ± 0.09	0.18 ± 0.11	0.43 ± 0.18			
Down						
All	0.16 ± 0.10	0.19 ± 0.13	0.43 ± 0.20*, **	<0.001	0.013	0.118
Male	0.19 ± 0.11	0.25 ± 0.17	0.55 ± 0.16			
Female	0.13 ± 0.09	0.14 ± 0.05	0.31 ± 0.16			

*Note*: Values are means ± standard deviations. Ten males and 10 females were included in the low frequency (LF) analysis, while data from one female participant is missing from the very low frequency (VLF) analysis. Point frequency estimates were used for RHE and SSM at 0.10 Hz and 0.05 Hz, while frequency bands were used for rest (LF: 0.05–0.15 Hz, VLF: 0.02–0.07 Hz). *p*‐values were calculated by mixed analysis of variance (ANOVA).

*p < 0.05 vs. rest, **p < 0.05 vs. RHE, ***p < 0.05: male vs. female.

Significant condition effects were found for all BEI measures (Table 2). In LF, overall BEI was higher during RHE and SSM than at rest, with the highest values during SSM (all *p* < 0.05). Up‐ and down‐BEIs were also higher during SSM compared to rest, with down‐BEI being higher in males than in females (all *p* < 0.05). In VLF, overall, up‐, and down‐BEIs were greater during SSM than during RHE and rest, with no significant sex differences.

### 3.6 Correlation analysis

**FIGURE 5:**
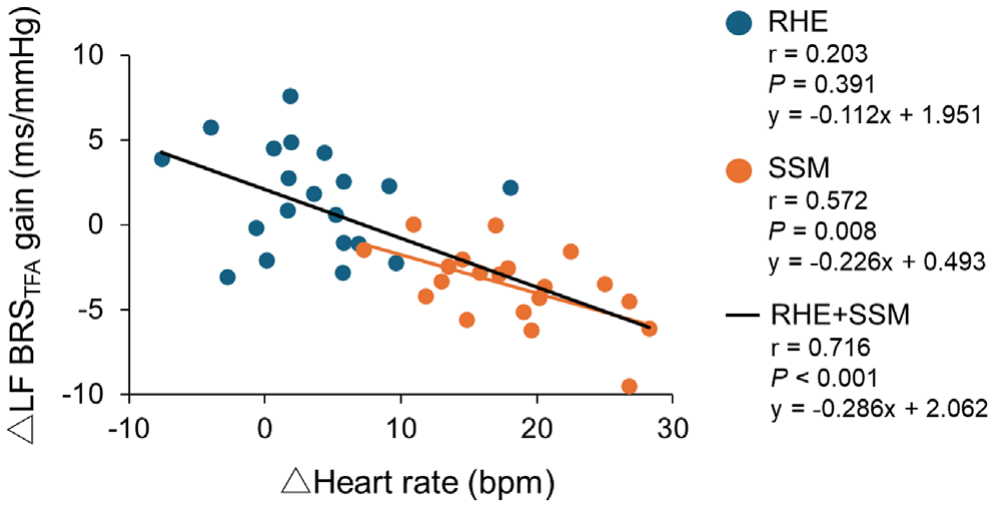
Correlation between individual changes in heart rate and cardiac baroreflex sensitivity calculated by transfer function analysis in low frequency (i.e., LF BRS_TFA_ gain). Individual changes were calculated by subtracting the RHE or SSM values from the resting values. Point estimate (0.10 Hz) is used for RHE and SSM, while frequency band (0.05–0.15 Hz) is used for spontaneous rest. Correlation coefficients and *p*‐values were calculated by the Pearson product–moment correlation analysis in 20 participants (10 males and 10 females). RHE: Repeated handgrip exercise, SSM: Sit‐stand maneuvers.

In LF, increased HR was associated with reduced BRS_TFA_ gain, particularly during SSM (Figure 5). Table 3 summarizes the correlations between BRS_TFA_ gain and BRS_SM_. During SSM, overall BRS_SM_ was correlated with BRS_TFA_ gains at both 0.10 and 0.05 Hz, although no significant correlations were found between BRS_TFA_ gain and overall BRS_SM_ in LF or VLF during rest or RHE.

**TABLE 3:** Simple correlations of baroreflex sensitivity analyzed by transfer function analysis (BRS_TFA_) and sequence method (BRS_SM_) in each condition.

BRS_TFA_		Overall BRS_SM_	Up‐BRS_SM_	Down‐BRS_SM_
Rest	LF band	−0.077 (0.748)	0.117 (0.632)	−0.199 (0.429)
	VLF band	0.216 (0.361)	0.592 (0.008)	−0.042 (0.869)
RHE	0.10 Hz	0.019 (0.935)	−0.224 (0.372)	0.289 (0.230)
	0.05 Hz	0.362 (0.128)	0.063 (0.797)	0.489 (0.039)
SSM	0.10 Hz	0.614 (0.004)	0.823 (<0.001)	0.167 (0.480)
	0.05 Hz	0.513 (0.021)	0.534 (0.015)	0.451 (0.053)

*Note*: Values are correlation coefficients and (*p*‐values) calculated by the Pearson product–moment correlation analysis in 20 participants.

Abbreviations: LF, low frequency; RHE, repeated handgrip exercise; SSM, sit‐stand maneuvers; VLF, very low frequency.

## 4 DISCUSSION

The main findings from this study are as follows. First, RHE increased SBP and HR oscillations, as quantified by PSD, with the highest PSD observed during SSM. Second, RHE significantly increased coherence for BRS_TFA_ estimation compared to spontaneous rest, with SSM exhibiting the highest coherence among all conditions. Third, BRS_TFA_ gain and BRS_SM_ remained unchanged during RHE and resting conditions but were reduced during SSM compared to RHE and rest. The reduction in BRS_TFA_ gain during 0.10 Hz SSM was associated with increased HR, suggesting baroreflex resetting during SSM. Fourth, males exhibited higher SBP oscillations during RHE in both LF and VLF than females, while BRS_TFA_ coherence was also greater in males during rest and LF RHE compared to females. Fifth, the correlations between BRS_TFA_ gain and BRS_SM_ were weak across all measured conditions. Collectively, these findings suggest that RHE can improve BRS_TFA_ estimation by increasing coherence compared to spontaneous rest, positioning RHE as an alternative for assessing cardiac BRS in individuals with physical limitations. Additionally, the weak correlations between BRS_TFA_ and BRS_SM_ imply that these BRS measures may capture different physiological aspects of cardiac BRS under different measurement conditions, such as RHE, SSM, and spontaneous rest.

Increased SBP fluctuation or direct stimulation of the arterial baroreceptor contributes to the reliable assessment of cardiac BRS. In this study, RHE significantly increased SBP fluctuation compared to spontaneous rest, although its magnitude was still lower than that during SSM. The difference in SBP fluctuation can be attributed to distinct physiological mechanisms involved in RHE and SSM. During RHE, SBP change is primarily driven by the exercise pressor reflex activating sympathetic neural activity (Kaufman & Hayes, 2002; Teixeira & Vianna, 2022). Repeated forearm contractions stimulate the mechanosensory reflex, while the accumulation of metabolic byproducts in the forearm activates the chemosensory reflex (Alam & Smirk, 1937, 1938). These reflexes increase sympathetic activity, leading to oscillatory SBP change during RHE. In contrast, SBP change during SSM is likely generated by both the exercise pressor reflex activated by lower‐body muscle contractions to change posture and the gravitational redistribution of blood volume (Hill, 1895). Transitioning from the seated to standing positions causes blood to pool in the lower limbs, reducing venous return, SV, and SBP (Blomqvist & Stone, 1991). In response, the cardiac baroreflex is activated to trigger feedback control of HR, while altered venous return (preload) may also influence HR through the cardiopulmonary baroreflex during SSM (Fadel & Raven, 2012). EtCO_2_ increased during SSM but not RHE, suggesting that the rise in EtCO_2_ is likely to reflect increased metabolic rate during repeated postural changes contracting lower‐body large muscles. This may have altered hemodynamic responses through the central chemoreflex. Therefore, the greater increase in SBP PSD during SSM compared to RHE and rest suggests the combined effects of exercise pressor reflex, gravity‐induced blood shift, central chemoreflex, and cardiopulmonary baroreflex.

Along with increased oscillations of SBP and RRI, coherence for BRS_TFA_ estimation increased during RHE and SSM compared to spontaneous rest. With TFA, coherence quantifies the strength of a correlation between input and output signals, while their magnitude and temporal relations are quantified by gain and phase (Ogoh et al., 2005). Thus, increased coherence during RHE and SSM suggests an improved signal‐to‐noise ratio for estimating BRS_TFA_, allowing baroreflex responses to be distinguished more effectively from background noise (Pinna et al., 2002). During spontaneous rest, the fluctuations of SBP and RRI are typically small, resulting in low coherence and less reliable estimation of cardiac BRS. To address this limitation, prior studies have employed methods such as the modified Oxford technique (Taylor et al., 2011), neck pressure chambers (Eckberg et al., 1975), and postural changes (Horsman et al., 2013; Zhang et al., 2009). While these methods increase SBP fluctuations, each often has drawbacks. The modified Oxford method is invasive and may not be suited for routine clinical assessment of BRS (Young et al., 2008). The use of neck pressure chambers is primarily limited to laboratory settings for assessing carotid baroreflex function (Fadel et al., 2003). Postural changes (e.g., SSM), although easy and non‐invasive, may be impractical for individuals with physical function impairments. Therefore, our results demonstrating that RHE performed at light intensity increases coherence for BRS_TFA_ estimation may offer a practical and non‐invasive alternative to existing methods, particularly for patients with lower‐body mobility limitations.

**FIGURE 6:**
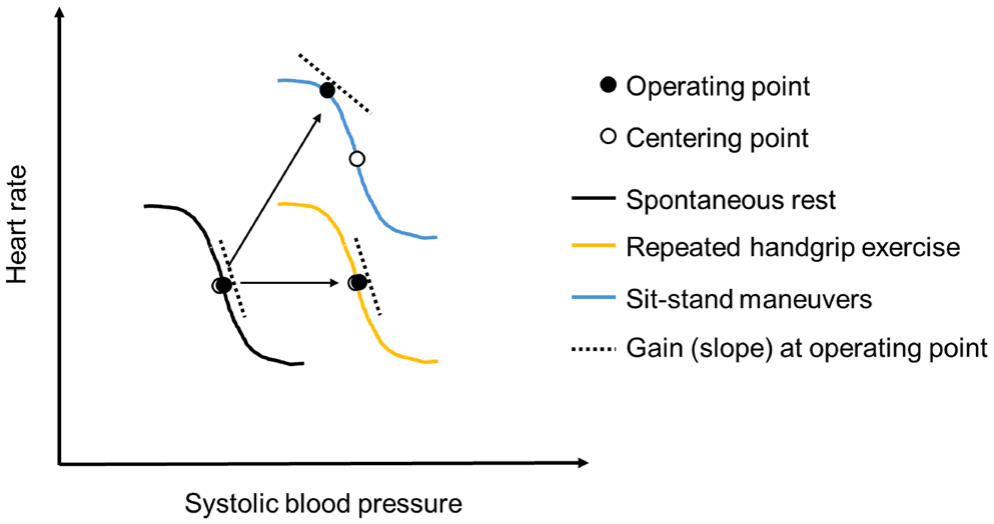
Hypothetical diagram illustrating the baroreflex curve shifts during repeated handgrip exercise and sit‐stand maneuvers compared to resting spontaneous condition.

BRS_TFA_ gain and BRS_SM_ were significantly lower during SSM at 0.10 Hz compared to RHE and rest within the same frequency range, while these BRS measures were similar during RHE and rest (Figure 4, Table 2). Additionally, during SSM, HR, SV, CO, and EtCO_2_ were significantly higher, while TPR was lower than during rest and RHE. Furthermore, reduced BRS_TFA_ gain during 0.10 Hz SSM was correlated with increased HR. Therefore, these results collectively suggest that SSM reset the baroreflex, likely due to altered autonomic neural function (i.e., vagal withdrawal and/or increased sympathetic activity) mediated through large‐muscle contractions during repeated postural changes (Ogoh et al., 2005) (Figure 6). During dynamic exercise like SSM, the baroreflex resetting may be characterized by the right upward shift of the SBP‐HR sigmoidal curve, with the operating point moving away from the centering point, consequently decreasing BRS (Ogoh et al., 2005). On the other hand, during RHE involving small muscle contractions, the baroreflex curve may be shifted rightward with increased SBP but no HR change.

Another potential mechanism by which SSM decreases BRS may be a limited time available for the baroreflex to respond during SSM at 0.10 Hz. Previous research showed that upon standing, HR and CO increase within ~3 s driven by central command and mechanoreceptor reflex (Horsman et al., 2014). Simultaneously, TPR decreases, causing ABP to drop despite the increased CO. Approximately 7 s after standing, ABP normalizes as baroreceptors unload, and sympathetic vasoconstriction restores total peripheral resistance. Thus, rapid postural changes during SSM at 0.10 Hz may not allow adequate baroreflex recovery and reduce its sensitivity. Additionally, SSM‐induced activation of group III/IV muscle afferents may contribute to carotid baroreflex resetting, which shifts the operating point without influencing spontaneous baroreflex responsiveness, thereby reducing BRS (Hureau et al., 2018).

During RHE at 0.10 Hz, BRS_TFA_ phase was greater than during spontaneous rest, indicating a longer delay of the HR response to SBP change. The latency of cardiac baroreflex is primarily determined by the vagal control of HR, which is faster than the sympathetic control (Eckberg & Sleight, 1992; Levy et al., 1993). Thus, increased sympathetic neural activity during RHE may have altered an autonomic balance to control HR, resulting in the increased phase lag of BRS_TFA_ during RHE at 0.10 Hz. Additionally, we observed that BRS_TFA_ gain was higher during SSM at 0.05 Hz than during spontaneous rest. The autonomic control of HR in VLF is governed by mechanisms that are less understood compared to LF (Hayano & Yuda, 2019), which complicates the interpretation of increased BRS_TFA_ during SSM at 0.05 Hz. One hypothesis would be that this increase resulted from metabolic or vagal effects on HR and ABP (Montano et al., 2009), although further investigation is needed to confirm the hypothesis.

Males had higher LF BRS_TFA_ coherence during both rest and RHE than females. This indicates a stronger correlation between the oscillations of SBP and HR in the LF range. Male participants also showed greater SBP oscillation during RHE than females, which may have enhanced the oscillatory synchronization between SBP and HR, thereby contributing to higher coherence in males. In contrast, females exhibited higher overall BRS_SM_ during RHE at 0.10 Hz, implying a higher vagally mediated baroreflex responsiveness to transient BP fluctuations. While these results differ from previous findings (Fu & Ogoh, 2019; Samora et al., 2019), they may be attributed to the small sample size and/or methodological variations in the current and previous study protocols, particularly the use of light‐intensity RHE.

## 5 METHODOLOGICAL CONSIDERATIONS

This study calculated cardiac BRS using the TFA and sequence methods in the frequency and time domains, respectively. With TFA, the entire data of continuous SBP and RRI signals are decomposed by the Fourier transform to provide sine waves in individual frequencies, followed by the estimations of magnitude (gain) and temporal (phase) relations. The reliability of TFA metric estimations depends on the coherence function, providing the signal‐to‐noise ratio for BRS_TFA_ estimation. On the other hand, BRS_SM_ is calculated from a part of the data where the consecutive beats of SBP and RRI change in the same direction. Using the sequence method, the overall and down‐BEIs increased during RHE in the LF band compared to spontaneous rest. This indicates the improved signal‐to‐noise ratio for BRS_SM_ estimation because BEI assesses how effectively the baroreflex translates SBP fluctuations into HR adjustments (Di Rienzo et al., 2001). Therefore, using both the TFA and sequence methods, our results suggest that RHE improves the estimations of cardiac BRS compared to spontaneous rest. However, the weak correlations between BRS_TFA_ and BRS_SM_ may be explained partly by the methodological differences, such as the use of entire or part of the data to quantify the dynamic SBP‐RRI relation (Laude et al., 2004).

This study has several limitations. The sequence method may not be very suited to estimate BRS in the resting and RHE conditions because small fluctuations of SBP result in fewer detectable sequences, potentially affecting the accuracy of BRS_SM_ estimation. The small sample size of healthy young adults also limits the generalizability and clinical applicability of our results to older adults and patients with chronic disease. The menstrual cycle phase was not controlled in female participants, which may have introduced confounding effects on outcomes.

Despite these limitations, our results suggest that light‐intensity RHE is a feasible option for assessing cardiac BRS in older adults or individuals unable to perform postural changes. The significant increase in coherence during RHE compared to spontaneous rest suggests improved BRS_TFA_ estimation. RHE may offer an advantage over SSM by not significantly changing HR, allowing reliable BRS assessment without baroreflex resetting. Our RHE protocol is also strengthened by using individually calibrated handgrip force relative to the maximal force and can be adjusted to various populations including healthy participants and patients. RHE introduces less noise in physiological waveform data due to less whole‐body movement than SSM. Lastly, our RHE protocol can be used to assess dynamic cerebral autoregulation simultaneously with cardiac BRS evaluation (Qin et al., 2024).

## 6 CONCLUSIONS

This study demonstrated that RHE performed at light intensity can significantly increase coherence and BEI for estimating cardiac BRS_TFA_ and BRS_SM_, respectively, accompanied by increased SBP and RRI oscillations in young healthy male and female participants. The BRS was similar during RHE and spontaneous rest. On the other hand, SSM generated the greatest SBP and RRI oscillations along with the highest BRS_TFA_ coherence, while BRS during 0.10 Hz SSM was significantly reduced and correlated with increased HR, which suggests baroreflex resetting due to the dynamic exercise effects of SSM. These findings indicate that RHE can improve cardiac BRS estimation and may offer a practical alternative for assessing BRS in individuals with physical limitations.

## AUTHOR CONTRIBUTIONS

The conception and design of the study were made by Takashi Tarumi. Wenxing Qin, Marina Fukuie, Daisuke Hoshi, and Shoya Mori collected and analyzed data. Wenxing Qin and Takashi Tarumi interpreted the results, prepared the figures and tables, and drafted the manuscript. Tsubasa Tomoto, Jun Sugawara, and Shigehiko Ogoh provided intellectual advice and revised the manuscript. The final version of the manuscript was approved by all authors.

## FUNDING INFORMATION

This study was supported by the Japan Society for the Promotion of Science (23K24748, 21K18299, 20H04086, 23KK0179). The ARIHHP Cooperative Grant (University of Tsukuba, TT). All authors report no disclosures relevant to this manuscript.

## ETHICS STATEMENT

The study complies with the standards set by the latest revision of the Declaration of Helsinki and was approved by the Institutional Review Boards of the National Institute of Advanced Industrial Science and Technology.

## Data Availability

The data that support the findings of this study are available from the corresponding author upon reasonable request.

## References

[phy270352-bib-0001] Aengevaeren, V. L. , Claassen, J. A. , Levine, B. D. , & Zhang, R. (2013). Cardiac baroreflex function and dynamic cerebral autoregulation in elderly masters athletes. Journal of Applied Physiology (1985), 114, 195–202. 10.1152/japplphysiol.00402.2012 23139365

[phy270352-bib-0002] Alam, M. , & Smirk, F. H. (1937). Observations in man upon a blood pressure raising reflex arising from the voluntary muscles. The Journal of Physiology, 89, 372–383. 16994867 10.1113/jphysiol.1937.sp003485 PMC1395054

[phy270352-bib-0003] Alam, M. , & Smirk, F. H. (1938). Observations in man on a pulse‐accelerating reflex from the voluntary muscles of the legs. The Journal of Physiology, 92, 167–177. 16994964 10.1113/jphysiol.1938.sp003592 PMC1395171

[phy270352-bib-0004] Bagnall‐Hare, H. , McLoone, V. I. , & Ringwood, J. V. (2024). On the accuracy of sequence methods for baroreflex sensitivity estimation. Physiological Engineering and Science in Medicine, 47, 503–516. 10.1007/s13246-023-01380-y PMC11166763 38564152

[phy270352-bib-0005] Blomqvist, C. G. , & Stone, H. L. (1991). Cardiovascular adjustments to gravitational stress. NASA Lyndon B Johnson Space Center, Spacelab Life Sciences 1: Reprints of Background Life Sciences Publications.

[phy270352-bib-0006] Dani, M. , Dirksen, A. , Taraborrelli, P. , Panagopolous, D. , Torocastro, M. , Sutton, R. , & Lim, P. B. (2021). Orthostatic hypotension in older people: Considerations, diagnosis and management. Clinical Medicine (London, England), 21, e275–e282. 34001585 10.7861/clinmed.2020-1044 PMC8140709

[phy270352-bib-0007] Di Rienzo, M. , Parati, G. , Castiglioni, P. , Tordi, R. , Mancia, G. , & Pedotti, A. (2001). Baroreflex effectiveness index: An additional measure of baroreflex control of heart rate in daily life. American Journal of Physiology. Regulatory, Integrative and Comparative Physiology, 280, R744–R751. 11171653 10.1152/ajpregu.2001.280.3.R744

[phy270352-bib-0008] Eckberg, D. L. , Cavanaugh, M. S. , Mark, A. L. , & Abboud, F. M. (1975). A simplified neck suction device for activation of carotid baroreceptors. The Journal of Laboratory and Clinical Medicine, 85, 167–173. 1141726

[phy270352-bib-0009] Eckberg, D. L. , & Sleight, P. (1992). Human baroreflexes in health and disease. Oxford University Press.

[phy270352-bib-0010] Fadel, P. J. (2008). Arterial baroreflex control of the peripheral vasculature in humans: Rest and exercise. Medicine & Science in Sports & Exercise, 40(12), 2055–2062. 10.1249/MSS.0b013e318180bc80 18981944

[phy270352-bib-0011] Fadel, P. J. , Ogoh, S. , Keller, D. M. , & Raven, P. B. (2003). Recent insights into carotid baroreflex function in humans using the variable pressure neck chamber. Experimental Physiology, 88, 671–680. 14603365 10.1113/eph8802650

[phy270352-bib-0012] Fadel, P. J. , & Raven, P. B. (2012). Human investigations into the arterial and cardiopulmonary baroreflexes during exercise. Experimental Physiology, 97, 39–50. 22002871 10.1113/expphysiol.2011.057554 PMC3253263

[phy270352-bib-0013] Fu, Q. , & Ogoh, S. (2019). Sex differences in baroreflex function in health and disease. The Journal of Physiological Sciences, 69, 851–859. 31721084 10.1007/s12576-019-00727-z PMC10717578

[phy270352-bib-0014] Hayano, J. , & Yuda, E. (2019). Pitfalls of assessment of autonomic function by heart rate variability. Journal of Physiological Anthropology, 38, 3. 30867063 10.1186/s40101-019-0193-2 PMC6416928

[phy270352-bib-0015] Herrmann, S. D. , Willis, E. A. , Ainsworth, B. E. , Barreira, T. V. , Hastert, M. , Kracht, C. L. , Schuna, J. M., Jr. , Cai, Z. , Quan, M. , Tudor‐Locke, C. , Whitt‐Glover, M. C. , & Jacobs, D. R., Jr. (2024). 2024 adult compendium of physical activities: A third update of the energy costs of human activities. Journal of Sport and Health Science, 13, 6–12. 38242596 10.1016/j.jshs.2023.10.010 PMC10818145

[phy270352-bib-0016] Hill, L. (1895). The influence of the force of gravity on the circulation of the blood. The Journal of Physiology, 18, 15–53. 10.1113/jphysiol.1895.sp000556 PMC1514613 16992266

[phy270352-bib-0017] Horsman, H. M. , Peebles, K. C. , Galletly, D. C. , & Tzeng, Y. C. (2013). Cardiac baroreflex gain is frequency dependent: Insights from repeated sit‐to‐stand maneuvers and the modified Oxford method. Applied Physiology, Nutrition, and Metabolism, 38, 753–759. 10.1139/apnm-2012-0444 23799277

[phy270352-bib-0018] Horsman, H. M. , Tzeng, Y. C. , Galletly, D. C. , & Peebles, K. C. (2014). The repeated sit‐to‐stand maneuver is a superior method for cardiac baroreflex assessment: A comparison with the modified Oxford method and Valsalva maneuver. American Journal of Physiology. Regulatory, Integrative and Comparative Physiology, 307, R1345–R1352. 25274908 10.1152/ajpregu.00376.2014

[phy270352-bib-0019] Hureau, T. J. , Weavil, J. C. , Thurston, T. S. , Broxterman, R. M. , Nelson, A. D. , Bledsoe, A. D. , Jessop, J. E. , Richardson, R. S. , Wray, D. W. , & Amann, M. (2018). Identifying the role of group III/IV muscle afferents in the carotid baroreflex control of mean arterial pressure and heart rate during exercise. The Journal of Physiology, 596, 1373–1384. 29388218 10.1113/JP275465 PMC5899981

[phy270352-bib-0020] Kaufman, M. P. , & Hayes, S. G. (2002). The exercise pressor reflex. Clinical Autonomic Research, 12, 429–439. 12598947 10.1007/s10286-002-0059-1

[phy270352-bib-0021] La Rovere, M. T. , Pinna, G. D. , & Raczak, G. (2008). Baroreflex sensitivity: Measurement and clinical implications. Annals of Noninvasive Electrocardiology, 13, 191–207. 18426445 10.1111/j.1542-474X.2008.00219.x PMC6931942

[phy270352-bib-0022] Laude, D. , Elghozi, J. L. , Girard, A. , Bellard, E. , Bouhaddi, M. , Castiglioni, P. , Cerutti, C. , Cividjian, A. , Di Rienzo, M. , Fortrat, J. O. , Janssen, B. , Karemaker, J. M. , Leftheriotis, G. , Parati, G. , Persson, P. B. , Porta, A. , Quintin, L. , Regnard, J. , Rudiger, H. , & Stauss, H. M. (2004). Comparison of various techniques used to estimate spontaneous baroreflex sensitivity (the EuroBaVar study). American Journal of Physiology. Regulatory, Integrative and Comparative Physiology, 286, R226–R231. 14500269 10.1152/ajpregu.00709.2002

[phy270352-bib-0023] Levy, M. N. , Yang, T. , & Wallick, D. W. (1993). Assessment of beat‐by‐beat control of heart rate by the autonomic nervous system: Molecular biology technique are necessary, but not sufficient. Journal of Cardiovascular Electrophysiology, 4, 183–193. 8269290 10.1111/j.1540-8167.1993.tb01222.x

[phy270352-bib-0024] Ma, Y. , Tully, P. J. , Hofman, A. , & Tzourio, C. (2020). Blood pressure variability and dementia: A state‐of‐the‐art review. American Journal of Hypertension, 33, 1059–1066. 32710605 10.1093/ajh/hpaa119

[phy270352-bib-0025] Ma, Y. , Zhang, Y. , Hamaya, R. , Westerhof, B. E. , Shaltout, H. A. , Kavousi, M. , Mattace‐Raso, F. , Hofman, A. , Wolters, F. J. , Lipsitz, L. A. , & Ikram, M. A. (2024). Baroreflex sensitivity and long‐term dementia risk in older adults. Hypertension, 82, 347–356. 39670317 10.1161/HYPERTENSIONAHA.124.24001 PMC11735285

[phy270352-bib-0026] Montano, N. , Porta, A. , Cogliati, C. , Costantino, G. , Tobaldini, E. , Casali, K. R. , & Iellamo, F. (2009). Heart rate variability explored in the frequency domain: A tool to investigate the link between heart and behavior. Neuroscience and Biobehavioral Reviews, 33, 71–80. 18706440 10.1016/j.neubiorev.2008.07.006

[phy270352-bib-0027] Ogoh, S. , Fisher, J. P. , Dawson, E. A. , White, M. J. , Secher, N. H. , & Raven, P. B. (2005). Autonomic nervous system influence on arterial baroreflex control of heart rate during exercise in humans. The Journal of Physiology, 566, 599–611. 15890708 10.1113/jphysiol.2005.084541 PMC1464761

[phy270352-bib-0028] Parati, G. , Casadei, R. , Groppelli, A. , Di Rienzo, M. , & Mancia, G. (1989). Comparison of finger and intra‐arterial blood pressure monitoring at rest and during laboratory testing. Hypertension, 13, 647–655. 2500393 10.1161/01.hyp.13.6.647

[phy270352-bib-0029] Pinna, G. D. , Maestri, R. , Raczak, G. , & La Rovere, M. T. (2002). Measuring baroreflex sensitivity from the gain function between arterial pressure and heart period. Clinical Science (London, England), 103, 81–88. 10.1042/cs1030081 12095408

[phy270352-bib-0030] Qin, W. , Fukuie, M. , Hoshi, D. , Mori, S. , Tomoto, T. , Sugawara, J. , & Tarumi, T. (2024). Dynamic cerebral autoregulation during repeated handgrip exercise: Comparisons with spontaneous rest and sit‐stand maneuvers. Journal of Applied Physiology (1985), 137(3), 718–727. 10.1152/japplphysiol.00217.2024 39116347

[phy270352-bib-0031] Raczak, G. , La Rovere, M. T. , Pinna, G. D. , Maestri, R. , & ŚWitecka, G. (2001). Assessment of baroreflex sensitivity in patients with preserved and impaired left ventricular function by means of the Valsalva manoeuvre and the phenylephrine test. Clinical Science, 100, 33–41. 11115415

[phy270352-bib-0032] Raven, P. B. , Fadel, P. J. , & Ogoh, S. (2006). Arterial baroreflex resetting during exercise: A current perspective. Experimental Physiology, 91, 37–49. 16210446 10.1113/expphysiol.2005.032250

[phy270352-bib-0033] Samora, M. , Teixeira, A. L. , Sabino‐Carvalho, J. L. , & Vianna, L. C. (2019). Spontaneous cardiac baroreflex sensitivity is enhanced during post‐exercise ischemia in men but not in women. European Journal of Applied Physiology, 119, 103–111. 30293100 10.1007/s00421-018-4004-y

[phy270352-bib-0034] Skow, R. J. , Garza, N. A. , Nandadeva, D. , Stephens, B. Y. , Wright, A. N. , Grotle, A. K. , Young, B. E. , & Fadel, P. J. (2022). Impact of COVID‐19 on cardiac autonomic function in healthy young adults: Potential role of symptomatology and time since diagnosis. American Journal of Physiology. Heart and Circulatory Physiology, 323, H1206–H1211. 36331556 10.1152/ajpheart.00520.2022 PMC9678405

[phy270352-bib-0035] Sleight, P. , La Rovere, M. T. , Mortara, A. , Pinna, G. , Maestri, R. , Leuzzi, S. , Bianchini, B. , Tavazzi, L. , & Bernardi, L. (1995). Physiology and pathophysiology of heart rate and blood pressure variability in humans: Is power spectral analysis largely an index of baroreflex gain? Clinical Science (London, England), 88, 103–109. 10.1042/cs0880103 7677832

[phy270352-bib-0036] Steptoe, A. , & Vogele, C. (1990). Cardiac baroreflex function during postural change assessed using non‐invasive spontaneous sequence analysis in young men. Cardiovascular Research, 24, 627–632. 2224929 10.1093/cvr/24.8.627

[phy270352-bib-0037] Sugawara, J. , Tanabe, T. , Miyachi, M. , Yamamoto, K. , Takahashi, K. , Iemitsu, M. , Otsuki, T. , Homma, S. , Maeda, S. , Ajisaka, R. , & Matsuda, M. (2003). Non‐invasive assessment of cardiac output during exercise in healthy young humans: Comparison between Modelflow method and doppler echocardiography method. Acta Physiologica Scandinavica, 179, 361–366. 14656373 10.1046/j.0001-6772.2003.01211.x

[phy270352-bib-0038] Tarumi, T. , & Zhang, R. (2023). Point‐counterpoint: Transfer function analysis of dynamic cerebral autoregulation: To band or not to band? Journal of Cerebral Blood Flow and Metabolism, 43, 1625–1627. 37303232 10.1177/0271678X231182245 PMC10414008

[phy270352-bib-0039] Taylor, C. E. , Atkinson, G. , Willie, C. K. , Jones, H. , Ainslie, P. N. , & Tzeng, Y. C. (2011). Diurnal variation in the mechanical and neural components of the baroreflex. Hypertension, 58, 51–56. 21632473 10.1161/HYPERTENSIONAHA.111.171512

[phy270352-bib-0040] Teixeira, A. L. , & Vianna, L. C. (2022). The exercise pressor reflex: An update. Clinical Autonomic Research, 32, 271–290. 35727398 10.1007/s10286-022-00872-3

[phy270352-bib-0041] Tomoto, T. , Repshas, J. , Zhang, R. , & Tarumi, T. (2021). Midlife aerobic exercise and dynamic cerebral autoregulation: Associations with baroreflex sensitivity and central arterial stiffness. Journal of Applied Physiology (1985), 131, 1599–1612. 10.1152/japplphysiol.00243.2021 PMC8616602 34647828

[phy270352-bib-0042] Young, C. N. , Fisher, J. P. , & Fadel, P. J. (2008). The ups and downs of assessing baroreflex function. The Journal of Physiology, 586, 1209–1211. 10.1113/jphysiol.2007.149484 PMC2375666 18187468

[phy270352-bib-0043] Zhang, R. , Claassen, J. A. , Shibata, S. , Kilic, S. , Martin‐Cook, K. , Diaz‐Arrastia, R. , & Levine, B. D. (2009). Arterial‐cardiac baroreflex function: Insights from repeated squat‐stand maneuvers. American Journal of Physiology. Regulatory, Integrative and Comparative Physiology, 297, R116–R123. 19420293 10.1152/ajpregu.90977.2008 PMC2711702

