# Correction to “Cardiac baroreflex sensitivity during repeated handgrip exercise: Comparisons with sit‐stand maneuvers and spontaneous rest”

**DOI:** 10.14814/phy2.70482

**Published:** 2025-07-26

Qin, W., Fukuie, M., Hoshi, D., Mori, S., Tomoto, T., Ogoh, S., Sugawara, J., & Tarumi, T. (2025). Physiological Reports, 13, e70352.

The authors' affiliations in the published article are incorrect. The correct affiliations are as follows:

Wenxing Qin^1,2^ | Marina Fukuie^2^ | Daisuke Hoshi^2^ | Shoya Mori^3^ | Tsubasa Tomoto^2^ | Shigehiko Ogoh^4,5^ | Jun Sugawara^2,3^ | Takashi Tarumi^2,3^

^1^Graduate School of Comprehensive Human Sciences, University of Tsukuba, Tsukuba, Ibaraki, Japan

^2^Human Informatics and Interaction Research Institute, National Institute of Advanced Industrial Science and Technology, Tsukuba, Ibaraki, Japan

^3^Institute of Health and Sport Sciences, University of Tsukuba, Tsukuba, Ibaraki, Japan

^4^Department of Biomedical Engineering, Toyo University, Saitama, Japan

^5^Neurovascular Research Laboratory, University of South Wales, Pontypridd, UK

We apologize for this error which does not affect the scientific content of the article.

